# Subjective Cognitive Complaints and the Role of Executive Cognitive Functioning in the Working Population: A Case-Control Study

**DOI:** 10.1371/journal.pone.0083351

**Published:** 2013-12-26

**Authors:** Cecilia U. D. Stenfors, Petter Marklund, Linda L. Magnusson Hanson, Töres Theorell, Lars-Göran Nilsson

**Affiliations:** 1 Department of Psychology, Stockholm University, Stockholm, Sweden; 2 Stress Research Institute, Stockholm University, Stockholm, Sweden; 3 Department of Public Health Sciences, Karolinska Institute, Stockholm, Sweden; University of Texas at Dallas, United States of America

## Abstract

**Background:**

Cognitive functioning is important for managing work and life in general. However, subjective cognitive complaints (SCC), involving perceived difficulties with concentration, memory, decision making, and clear thinking are common in the general and working population and can be coupled with both lowered well-being and work ability. However, the relation between SCC and cognitive functioning across the adult age-span, and in the work force, is not clear as few population-based studies have been conducted on non-elderly adults. Thus, the present study aimed to test the relation between SCC and executive cognitive functioning in a population-based sample of employees.

**Methods:**

Participants were 233 employees with either high (cases) or low (controls) levels of SCC. Group differences in neuropsychological test performance on three common executive cognitive tests were analysed through a set of analyses of covariance tests, including relevant covariates.

**Results & Conclusions:**

In line with the a priori hypotheses, a high level of SCC was associated with significantly poorer executive cognitive performance on all three executive cognitive tests used, compared to controls with little SCC. Additionally, symptoms of depression, chronic stress and sleeping problems were found to play a role in the relations between SCC and executive cognitive functioning. No significant associations remained after adjusting for all these factors. The current findings contribute to an increased understanding of what characterizes SCC in the work force and may be used at different levels of prevention of- and intervention for SCC and related problems with executive cognitive functioning.

## Introduction

Self-reported problems with cognitive functioning, such as difficulties with concentration, memory, decision making, and clear thinking, are common in the general and working population. We will refer to these as subjective cognitive complaints (SCC), although many different terms are being used in the literature. SCC often co-occur with other common psychological health problems that belong to the major causes of sick leave [1], including depression, chronic stress/exhaustion and sleeping problems, and are also related to lower quality of life [2-5]. SCC in the frame of depressive and stress related conditions have also been described as a key factor that reduce work ability even in phases that are not characterised by clinical illness (e.g. 6,7). SCC may, thus, indicate actual cognitive deficits that require appropriate aids or interventions to handle or improve functionality. 

The main part of previous research on the relation between SCC and cognitive functioning has been performed on elderly people in the context of cognitive aging and dementia. Here, the validity of SCC as an indicator of actual cognitive deficits has been questioned as some have found no [8,9], or weak relationships between the two [10,11]. Other recent research on SCC utilising longitudinal designs and/or brain imaging have supported relationships between SCC and actual cognitive decline [12,13], as well as underlying functional and structural brain changes or Alzheimer’s disease pathology [14-18] .

Still, however, the relation between SCC and actual deficits in cognitive functioning has not been clearly established and particularly few studies exist on non-clinical general working populations. Hence, this is the focus of the present study. 

SCC in non-elderly has been tested and found by some to be related to poorer executive functioning [19,20], and to poorer episodic memory functioning [19-21]. Others have found little association between SCC and cognitive function [2,22], although the subjective and objective cognitive measures were more limited in one of those studies [22].

In our own recent study of the relations between SCC and declarative memory functioning, memory performance in an immediate free recall task was found to be selectively poorer under conditions of high executive demands (divided attention conditions) among those with a high degree of SCC, while only a trend was seen towards poorer ability on other episodic memory tasks (delayed recall of words and face recognition) and no deficits were seen in semantic memory functioning (C. Stenfors et al. 2013, submitted manuscript under review). This suggests that SCC in the general working population may be related to executive functioning like processes in working memory (WM), attentional monitoring and flexibility. 

Modern work life may place a particularly high load on executive functions in day-to-day work, due to for example intensive and multiple information and communication channels that may create a scattered psychosocial work environment [23]. Lower levels of executive functioning may therefore also be particularly noticeable to working individuals. These functions are important in managing every-day life in general, including executive tasks such as planning and problem solving, and are also important for self-regulation, emotional regulation and coping with stress [24-26]. Cognitive processes involved in flexibility and WM- including attentional shifting and maintenance, updating and manipulation of information in awareness/in the focus of attention- heavily depend on prefrontal cortical (PFC) brain regions, prefrontal-parietal networks and the hippocampus [27-33] . These functions and structures also seem to be particularly vulnerable to other psychological problems, i.e. symptoms of chronic stress, depression and sleeping problems, that often co-occur with SCC in the general population, as mentioned [2,3,5]. Executive functions like WM and attentional shifting seem to be particularly vulnerable to detrimental effects from both acute [34-39] and chronic [40-44] stress exposures, which may be particularly relevant when considering SCC among individuals in the work force. Studies of clinical groups with chronic stress conditions have showed that these patients often suffer from pronounced SCC with poorer cognitive functioning in episodic memory and executive functioning [42,43,45] 

Furthermore, executive cognitive deficits are also implicated in depressive conditions [46], as well as in poor sleep [47].

Cognitive problems in the general working population, as in these groups, may stem from psychobiological processes causing suboptimal conditions for executive cognitive functioning, rather than dementia pathology. This is also in line with the subjective experience of younger adults who themselves tend to ascribe their SCC to causes like stress, tension and emotional problems [4,5]. 

Taken together these observations give reasons to expect that SCC in the general working population may be associated with executive cognitive functioning and thus, the aim of the present case-control study was to test this.

It was predicted that a high- compared to a low level of reported SCC would be associated with poorer performance on neuropsychological test measures of working memory functioning and attentional shifting, while no differences between the SCC groups were expected on measures which do not place high demands on executive cognitive processes but rather tap perceptual- and motor speed.

Due to the observed overlap of SCC with depressive symptoms, chronic stress symptoms and sleeping problems, additional analyses testing the role of these factors were also planned. It was predicted that each of these factors would reduce the magnitude of any relationships between SCC and measures of executive cognitive functioning.

## Methods

### Participants and Study design

Participants were recruited from the 2010 wave of the Swedish Longitudinal Occupational Survey of Health (SLOSH)- a longitudinal study of work life, social situation and health among Swedish employees, conducted biennially. 

The SLOSH 2010 sample is based on the respondents to the nationally representative Swedish Work Environment Surveys (SWES) conducted biennially on the work force. (See, e.g. 48,49). Participants from SWES 2003, 2005 and 2007, 16-64 years of age at the time of the surveys, were asked to participate in the SLOSH wave 2010.

A total of 11525 subjects participated (57%) in SLOSH 2010 and 9132 of the participants were still gainfully employed at the time of participation in SLOSH 2010- i.e. they were in gainful employment during the past three months at a level of 30% of full time or more. 

Gainfully working participants in the Stockholm county and the counties surrounding the city of Gothenburg were then invited to participate in the present nested case-control study of cognitive functioning, based on their recently reported levels of SCC in the SLOSH 2010 questionnaire.

Case- and control groups with high versus low levels of SCC were defined and selected based on the levels of SCC reported in the SLOSH 2010 questionnaire. SCC were measured by a scale consisting of four questions about difficulties during the past 3 months with concentration, memory, decision-making, and ability to think clearly, adopted from the Copenhagen Psychosocial Questionnaire [50] originally from The Stress Profile questionnaire [51]. The mean score of the four items was used as a global measure of SCC for classifying subjects into high versus low levels of SCC. The scale has high internal consistency (Cronbach’s alpha=0.91). 

Those reporting SCC at a mean level of ≥3.25 were defined as cases, with a “high” level of SCC. This score corresponds to reporting that at least one of the four cognitive problems were experienced “Often” or more, and the other three problems were experienced at least “Sometimes”. This cut-off for the case group was based on face validity and on the distribution of SCC in the gainfully working part of the SLOSH population (8943 people), where a SCC score ≥3.25 corresponds to approximately the top decile of the distribution of SCC. The experimental control group on the other hand consisted of people with a reported “low” level of SCC, defined as a SCC score ≤2.0.This corresponds to experiencing the four cognitive problems “Seldom” or less on average, and belongs to approximately the bottom 50 % of the distribution of SCC scores. Controls were matched to the cases on geographical area, age, sex, and educational level.

All 352 identified cases and 941 case-matched controls were invited. More controls were invited in order to increase the possibility of getting matching controls for each case deciding to participate. 

Participants were invited consecutively to the present study within 1-3 weeks of responding to the SLOSH questionnaire.

Those consenting to participate were then given an appointment in Stockholm or Gothenburg for neuropsychological testing which occurred within approximately 4-16 weeks of responding to the SLOSH 2010 questionnaire. All the neuropsychological assessments for any individual participant were performed on the same occasion. The neuropsychological assessments were performed for all participants during three consecutive months and both cases and controls were tested across this three month period. However, on average, cases were tested 24 days earlier than controls during this period. 

A total of 233 participants took part in the study, out of which 116 (30 men, 86 women) were cases, and 117 (26 men, 91 women) were controls. 

Participants with any known possible brain injury, such as prior head trauma, stroke, or chemical poisoning, as well as psychotic illness, or other illness conditions at the time of testing were excluded from the study. Seven individuals were excluded from the study for such reasons. The sample of eligible participants thus consisted of 112 cases and 114 controls.

Cases were 25-67 years and controls were 29-66 years of age. Sample characteristics of the case and control groups are shown in table 1.

**Table 1:** Characteristics of groups with a low vs. high level of SCC.

*Measure* (*scale*)	Low SCC				High SCC				t-test	Persons chi^2^
	N	% within Low SCC	Mean	SD	n	% within High SCC	Mean	SD	t ^sign. level^	Chi^2 sign. level^
N	114	100	∙	∙	112	100	∙	∙	∙	∙
Sex:	∙	∙	∙	∙	∙	∙	∙	∙	∙	.53
*Male*	26	22.8	∙	∙	27	24.1	∙	∙	∙	∙
*Female*	88	77.2	∙	∙	85	75.9	∙	∙	∙	∙
Age	114	∙	48.66	10.08	112	∙	48.69	10.66	-.02	∙
Education:	∙	∙	∙	∙	∙	∙	∙	∙	∙	1.98
*Upper secondary or lower*	37	32.5	∙	∙	40	35.7	∙	∙	∙	
*Univ.studies < 2 years*	9	7.9	∙	∙	14	12.5	∙	∙	∙	
*Univ. studies ≥ 2 years*	68	59.6	∙	∙	58	51.8	∙	∙	∙	
Yearly income (1000’s SKR)	114	∙	389.79	190.94	112	∙	334.82	153.99	2.38*	
SCC (1-5)	114	∙	1.56	.39	112	∙	3.72	.47	-37.80***	
Emotional exhaustion index (1-6)	114	.	1.66	0.79	111	.	3.56	1.34	-12.89***	
Depressive symptoms index (1-5)	114	∙	1.49	0.56	111	∙	3.16	1.04	-14.98***	
Disturbed sleep, prevalence	8	7	∙	∙	59	52.7	∙	∙	∙	60.50***
Awakening problems , prevalence	20	17.5	∙	∙	57	50.9	∙	∙	∙	29.76***
CVD, prevalence	3	2.6	∙	∙	6	5.4	∙	∙	∙	1.10
Diabetes, prevalence	3	2.6	∙	∙	4	3.6	∙	∙	∙	.17
Non-specific psych. illness, prevalence	1	0.9	∙	∙	15	13.4	∙	∙	∙	13.76***
SMBQ**^*†*^**	114	∙	2.33	0.94	112	∙	4.50	1.33	-14.12***	∙
*Mental fatigue/ cognitive subscale*	113	∙	2.04	0.91	112	∙	4.50	1.40	-15.69***	∙
Depressive symptoms index **^*†*^**	114	∙	1.55	0.64	112	∙	3.40	0.71	-12.51***	∙
MDI score**^*†*^**	114	∙	4.78	4.59	112	∙	16.71	10.55	-10.99***	∙
*Mild, prevalence*	1	0.9	∙	∙	14	12.5	∙	∙	∙	∙
*Moderate, prevalence*	1	0.9	∙	∙	14	12.5	∙	∙	∙	∙
*Severe, prevalence*	0	0	∙	∙	14	12.5∙	∙	∙	∙	∙

SMBQ= Shirom Melamed burnout questionnaire; MDI= major depression inventory.

^†^ Collected at the laboratory test occasion.

*p<0.05 **p<0.01 ***p<0.001

### Neuropsychological tests of executive functioning

Neuropsychological assessments were performed by a psychologist in a laboratory.

WM was measured using two classical WM tasks, a *reading span task* [52] and a verbal *2-back task* [53] on a computer. Both these tests were adopted from the Betula Study [54,55].

Attentional set-shifting was measured using the trail-making test (TMT) A and B [56]. 

**The reading span task** used was a modified version of the original test [52] with recommended modifications [57,58]. This test requires the simultaneous maintenance of to-be-remembered items and processing of other information and measures verbal working memory capacity.

In this test, the participant is shown a set of sentences, each followed by a word to be remembered, on a computer screen. The participant is required to read aloud the sentence on the screen and answer ”yes” or ”no” aloud and by pressing a Yes or No key, as to whether the sentence is correct or nonsense. Then the participant is required to read the single word aloud that is presented after the sentence and try to remember this word in the correct serial position for immediate recall after all sentences and words in the trial have been presented. Recalled items at the end of each trial are registered by the test administrator.

A trial contains 2, 3, 4 or 5 sentence-word pairs, i.e. there are 4 different levels of WM load, or difficulty, among trials (level 2-5).

The test contains 16 trials in total, 4 trials at each load level, and trials at different load levels are presented in randomized order to avoid the progressive build-up of proactive interference effects at higher load levels.

Two trials (at level 2) were given as training to ensure that the participant had understood the task correctly.

The test measures of verbal WM capacity used were [59]: 1) Span level. This is estimated by taking the load level at which the subject can perform ¾ of the trials perfectly, i.e. recall all words in at least ¾ of the trials correctly and in the correct serial order. Added to this number is the proportion of words recalled correctly at the next level. 2) The total number of correctly freely recalled words over all trials, regardless of correct recall of serial position order.

**The 2-back task** taps the WM processes of maintenance and updating. 

In this test, single words are shown on a computer screen consecutively and the task is to answer for each word whether it is the same as the word shown 2 items back by pressing the ”Yes”- or the ”No” key. There were 40 items (consecutive words) in total. Of these, 22.5 % (9/40) items were ”Hit/target items” ( i.e. the word shown is similar to the word 2 items back and the correct response is ”Yes”), 22.5 % (9/40) were target stimuli items (i.e. the stimulus word is new and the same as the word that will be shown 2 items forward), 20 % (8/40) were 3-back items (i.e. the stimulus word is the same as that which was shown 3 items back). Another 20 % (8/40) were 3-back stimulus words (i.e. the stimulus word is new and the same as the word that will be shown 3 items forward). Still another 5% (2/40) were 4-back items, and 5% were 4-back stimulus items, and 5 % (2/40) were new words shown only once in the test session. The trials with 3-back and 4-back items serve as lures, as the participant has to distinguish between recognised items in terms of their order in the stream of stimuli and answer “No” for all items that have been seen if they were not seen exactly 2 trials back.

The stimulus/word presentation duration was 2500 ms and the intertrial interval was 2000 ms. 

A block of 15 items were given as training in order for the participant to understand the task correctly, using both target items that required a “yes” response, as well as new and lure items that required a “no” response.

As there are multiple measures from the 2-back task that are being used in the literature, several measures were included also in the present study, in order to give a more full account of test performance. The test measures used were the 1) mean response accuracy, 2) mean response time (RT), and 3) “inverse efficiency” (IE) RT, on a) all 40 trials, b) trials with new word stimuli, c) trials with 2-back/target stimuli, and d) trials with 3-back lure stimuli. IE´s were computed by dividing the response time by the accuracy, for each specified category of items respectively (i.e. a-d) (adopted from [60]). By adjusting the response time as a function of the level of accuracy of responses, the inverse efficiency gives a measure of performance efficiency in the 2-back task. Since there are only 2 trials in the test that are 4-back lures, separate measures on those trials are not presented. 

Responding on the 2-back target trials and the 3-back lures is more difficult as they require the ability to discriminate an item in a target position from items in other list positions. New items, in contrast, can be rejected on the basis of an overall assessment of familiarity rather than the retrieval of positional information. Assuming that correct rejection of new items depend on the ability to use the low familiarity value, while correct responding on 2-back target trials and 3-back lures also depend heavily on the ability to use positional information, it is logical to analyze both these trial types separately to investigate updating ability specifically [53]. That is, the latter type of trials entail additional cognitive demands on updating of temporal/order information and are generally more difficult. Thus, we predicted that accuracy will be lower, response times longer and IE’s longer in 2-back target trials as well as 3-back lures among participants with a high level of SCC compared to those with a low level of SCC, while no group differences were expected on the “easy” trials with new stimuli words. 

**The trail-making tests (TMT**)** A and B** assess psychomotor *speed* and *attention al shifting* [56]. TMT A is a baseline test condition where the task is to connect, by making pencil lines, 25 encircled numbers randomly arranged on a page in proper order. Part A gives a baseline measure of perceptual processing and motor speed. TMT B additionally has an attentional shifting component where the task is instead to connect 25 encircled numbers and letters in alternating order. Accuracy and response times are measured. The main test measures are the response times in each part and the difference/cost incurred by the shifting component in part B relative to part A (i.e. response time on part B minus response time on part A).

### Other self-rated measures from the SLOSH 2010 questionnaire

Other questionnaire measures included scales on chronic stress, depressive symptoms and sleeping problems, for which high values on any measure indicate a high level of the construct.

**Chronic stress symptoms** were measured by the Maslach Burnout Inventory General Survey, using the subscale of *emotional exhaustion* measured by 5 items on a scale of 1-6/’A few times a year or less’-‘Every day’. This subscale has been proved to be the most robust and reliable [61,62]. Mean scores were used.

**Depressive symptoms** were measured by six items (scale 1-5) selected from the Hopkins Symptom Checklist core depression subscale (SCL-CD_6_, [63]). 

Mean scores were used.

**Sleeping problems:** The established and validated measures Disturbed sleep index (DSI) reflecting lack of sleep continuity and the Awakening index (AI) reflecting feelings of being insufficiently restored during the past 3 months, were used. Dichotomised variables were used indicating the presence or absence of sleep disturbances and awakening problems, based on four and three items respectively [64-66].

### Other potential confounders considered

Age, Sex, Educational level achieved (‘Upper secondary school or lower’, ‘Undergraduate studies <2 years’, ‘Undergraduate studies >2 years); yearly income from work; and the presence of cardiovascular disease, diabetes or (unspecific) psychiatric illness. 

### Additional self-rating measures from the laboratory test occasion

**Major depression inventory** (MDI), measuring the degree of depressive symptoms during the past two weeks with 10 items rated on a scale of 0-5 [67]. The sum of item scores (0-50) were used and indicate the degree of depressive symptoms as none (0-23), mild (20-24), moderate (25-29), or severe (≥30).

**Shirom Melamed Burnout Questionnaire** (SMBQ): a 22 item measure rated on a scale of 1-7. The mean scores were used as indicators of the degree of chronic stress and burnout symptoms experienced during the past month [68,69]. The questionnaire also includes a subscale on mental fatigue/cognitive symptoms which resemble the SCC items on problems concentrating or thinking clearly. Descriptive statistics are also presented for this subscale in table 1.

### Data analysis

Differences in WM and TMT test measures between groups with a high versus a low level of SCC were analysed using a set of separate Analysis of Covariance (ANCOVA) tests, adjusting for age, gender and education by adding these as covariates in the analysis. The other variables listed under “other potential confounders” above were not included in the analyses as these were not bivariately correlated with SCC and the cognitive measures.

Test scores potentially affected by poor vision and poor abilities in the Swedish language were excluded. 

Additional ANCOVAs were performed to test the role of depressive symptoms, chronic stress symptoms and sleeping problems in any relations between SCC level and the executive cognitive measures.

As stated, multiple analyses of group differences were performed, in favour of a detailed picture of cognitive test performances in the two SCC groups. Since the tests were planned a priori with clear hypotheses regarding the results, and since the cognitive test measures are inter-related, adjustments of the alpha levels according to the total number of group comparisons were not applied.

Data analyses were performed using SPSS 21 software.

### Ethics statement

The study has been approved by the Regional Research Ethics Board in Stockholm (Dnr 2010/397-31).

All study participants have given their written informed consent. Data were analysed anonymously.

## Results

Characteristics of the study groups with a high versus a low level of SCC are presented in table 1, including the prevalence of other psychological symptoms and medical conditions and results from t-tests and Chi^2^ tests of group differences on those measures. Individuals with a high level of SCC had significantly more symptoms of depression, chronic stress and sleeping problems, both at the time of responding to the SLOSH questionnaire and at the time of neuropsychological assessment shortly thereafter. Furthermore, the high SCC group also had significantly higher scores than the low SCC group on the cognitive subscale of the SMBQ at the test occasion. These scores were also highly correlated (r=0.71, p<0.000) with the SCC scores measures by the SLOSH questionnaire prior to the neuropsychological test occasion.

Descriptive statistics for the executive cognitive test measures by SCC group are shown in table 2. Results from the main ANCOVA tests of group differences in performance on the three executive cognitive tests are shown in table 3. Results after additional adjustments for symptoms of chronic stress, depression and sleeping problems are shown in table 4.

**Table 2:** Descriptive statistics for test performance measures in groups with low versus high levels of SCC.

Measure	Low SCC			High SCC		
	n	Mean	SD	n	Mean	SD
**Reading span**						
*Span level*	112	2.67	0.67	104	2.35	0.63
*Total free recall correct*	113	36.38	6.51	107	33.66	6.36
**2-back, response accuracy**						
*Overall*	113	0.88	0.08	109	0.86	0.09
*New words*	113	0.99	0.03	109	0.99	0.02
*2-back/target trials*	113	0.86	0.15	109	0.82	0.17
*3-back lures*	113	0.64	0.26	109	0.60	0.28
**2-back, response time**						
*Overall*	113	1131.21	190.21	109	1123.72	164.92
*New words*	113	1063.99	241.75	109	1020.18	212.29
*2-back/target trials*	113	1076.20	222.62	109	1144.71	235.88
*3-back lures*	113	1336.69	237.02	109	1311.47	231.47
**2-back, “inverse efficiency”**						
*Overall*	113	1303.67	269.76	109	1324.26	272.07
*New words*	113	1288.87	477.51	109	1313.98	498.03
*2-back/target trials*	113	1335.96	642.07	109	1502.51	604.97
*3-back lures*	113	2786.68	2281.68	109	3170.91	2752.72
**TMT, response time**						
*Part A*	113	26.50	7.81	111	26.62	8.36
*Part B*	113	56.54	23.06	111	66.23	31.94
*Difference (B-A)*	113	30.04	20.58	111	39.60	29.27

**Table 3:** Test results of group differences in cognitive performance domains between low vs. high SCC groups**†**.

*Dependent measure*	Source	d.f.	SS	MS	*F*	*p*	*η_p_^2^*
**Reading span**							
*Span level*	Group	1	5.038	5.038	**12.848**	**0.000**	**0.057**
	Error	211	82.738	0.392	**.**	**.**	**.**
*Total free recall correct*	Group	1	374.975	374.975	**9.968**	**0.002**	**0.044**
	Error	215	8087.604	37.617	.	.	.
**2-back, accuracy**							
*Overall*	Group	1	0.014	0,014	2.316	0.130	0.011
	Error	217	1.290	0,006	.	.	.
*New stimuli/words*	Group	1	0.001	0,001	0.779	0.379	0.004
	Error	217	0.172	0,001	.	.	.
*2-back/target trials*	Group	1	0.106	0,106	**4.367**	**0.038**	**0.020**
	Error	217	5.249	0,024	.	.	.
*3-back lure trials*	Group	1	0.082	0,082	1.284	0.258	0.006
	Error	217	13.933	0,064	.	.	.
**2-back, RT’s**							
*Overall*	Group	1	1661.76	1661,76	0.053	0.819	0.000
	Error	217	6860438.836	31614,926	.	.	.
*New words/stimuli*	Group	1	92770.00	92770,00	1.794	0.182	0.008
	Error	217	11222351.79	51715,907	.	.	.
*2-back/target trials*	Group	1	260194.20	260194,20	**5.126**	**0.025**	**0.023**
	Error	217	11014990.95	50760,327	.	.	.
*3-back lure trials*	Group	1	26365.24	26365,24	0.485	0.487	0.002
	Error	217	11784985.66	54308,690	.	.	.
**2-back, IE’s**							
*Overall*	Group	1	25756.47	25756.47	0.364	0.547	0.002
	Error	217	15346945.13	70723.25	.	.	.
*New words/stimuli*	Group	1	45185.45	45185.45	0.190	0.664	0.001
	Error	217	51734233.31	238406.61	.	.	.
*2-back/target trials*	Group	1	1534328.41	1534328.41	**4.019**	**0.046**	**0.018**
	Error	217	82841410.90	381757.65	.	.	.
*3-back lure trials*	Group	1	6429813.78	6429813.78	1.134	0.288	0.005
	Error	217	1230793775.46	5671860.72	.	.	.
**TMT**							
*Part A*	Group	1	0.26	0.26	0.005	0.945	.000
	Error	219	11823.44	53.99	.	.	.
*Part B*	Group	1	4603.73	4603.73	**6.861**	**0.009**	**.030**
	Error	219	146949.15	671.00	.	.	.
*Difference (B-A)*	Group	1	4534.85	4534.85	**7.673**	**0.006**	**.034**
	Error	219	129437.75	591.04	.	.	.

^†^ Using one-way between-subjects ANCOVAs with covariates age, sex and education.

d.f.=degrees of freedom. SS=sum of squares. MS=mean square.

**Table 4:** ANCOVA results for SCC group differences in executive cognitive measures, additionally controlling for symptoms of exhaustion, depression and sleeping problems in models a-d^†^.

Measure	Model	Source	df	Type III Sum of Squares	Mean Square	F	p-value	Partial Eta Squared
**RS task**								
Span level	a	SCC Group	1	1.07	1.07	2.79	**0.096**	**0.013**
		Error	209	79.75	0.38	.	.	.
Span level	b	SCC Group	1	1.90	1.90	4.80	**0.030**	**0.022**
		Error	209	82.66	0.40	.	.	.
Span level	c	SCC Group	1	2.21	2.21	5.55	**0.019**	**0.026**
		Error	204	81.29	0.40	.	.	.
Span level	d	SCC Group	1	0.72	0.72	1.83	0.178	0.009
		Error	200	78.54	0.39	.	.	.
FRC	a	SCC Group	1	68.99	68.99	1.88	0.171	0.009
		Error	213	7804.25	36.64	.	.	.
FRC	b	SCC Group	1	59.16	59.16	1.57	0.211	0.007
		Error	213	8014.83	37.63	.	.	.
FRC	c	SCC Group	1	117.21	117.21	3.15	**0.077**	**0.015**
		Error	208	7741.45	37.22	.	.	.
FRC	d	SCC Group	1	17.25	17.25	0.47	0.494	0.002
		Error	204	7510.48	36.82	.	.	.
**2-back task**								
Response accuracy on target/hit trials	a	SCC Group	1	0.02	0.02	0.80	0.372	0.004
		Error	215	5.21	0.02	.	.	.
Response time on target/hit trials	a	SCC Group	1	118232.75	118232.75	2.31	0.130	0.011
		Error	215	10993720.09	51133.58	.	.	.
IE on target/hit trials	a	SCC Group	1	41143.42	41143.42	0.11	0.742	0.001
		Error	215	81589722.79	379487.08	.	.	.
Response accuracy on target/hit trials	b	SCC Group	1	0.03	0.03	1.34	0.248	0.006
		Error	215	5.24	0.02	.	.	.
Response time on target/hit trials	b	SCC Group	1	2029.64	2029.64	0.04	0.840	0.000
		Error	215	10656803.63	49566.53	.	.	.
IE on target/hit trials	b	SCC Group	1	160742.99	160742.99	0.42	0.517	0.002
		Error	215	82193180.28	382293.86	.	.	.
Response accuracy on target/hit trials	c	SCC Group	1	0.03	0.03	1.41	0.236	0.007
		Error	209	5.08	0.02	.	.	.
Response time on target/hit trials	c	SCC Group	1	161174.65	161174.65	3.17	**0.077**	**0.015**
		Error	209	10634653.88	50883.51	.	**.**	**.**
IE on target/hit trials	c	SCC Group	1	316691.64	316691.64	0.83	0.364	0.004
		Error	209	79977881.13	382669.29	.	.	.
Response accuracy on target/hit trials	d	SCC Group	1	0.01	0.01	0.41	0.521	0.002
		Error	205	5.04	0.03	.	.	.
Response time on target/hit trials	d	SCC Group	1	2.90	2.90	0.00	0.994	0.000
		Error	205	10165493.95	49587.78	.	.	.
IE on target/hit trials	d	SCC Group	1	899.89	899.89	0.00	0.961	0.000
		Error	205	78809537.05	384436.77	.	.	.
**TMT**								
TMT B	a	SCC Group	1	116.89	116.89	0.18	0.674	0.001
		Error	217	142957.57	658.79	.	.	.
TMT B-A difference	a	SCC Group	1	99.87	99.87	0.17	0.678	0.001
		Error	217	125546.67	578.56	.	.	.
TMT B	b	SCC Group	1	131.79	131.79	0.20	0.657	0.001
		Error	217	144267.62	664.83	.	.	.
TMT B-A difference	b	SCC Group	1	29.55	29.55	0.05	0.822	0.000
		Error	217	125895.69	580.16	.	.	.
TMT B	c	SCC Group	1	1966.77	1966.77	2.86	**0.092**	**0.013**
		Error	211	144897.89	686.72	.	**.**	**.**
TMT B-A difference	c	SCC Group	1	2535.51	2535.51	4.23	**0.041**	**0.020**
		Error	211	126370.93	598.91	.	**.**	**.**
TMT B	d	SCC Group	1	6.40	6.40	0.01	0.923	0.000
		Error	207	141580.51	683.96	.	.	.
TMT B-A difference	d	SCC Group	1	7.49	7.49	0.01	0.911	0.000
		Error	207	122789.10	593.18	.	.	.

^†^ All models include the covariates age, sex and education.

^a^ Chronic stress symptoms measure is included as a covariate.

b. Depressive symptoms measure is included as a covariate.

^c^ Measures of sleeping problems are included as covariates.

^d^ Measures of chronic stress symptoms, depressive symptoms and sleeping problems are included as covariates.

RS=reading span task; FRC=free recall, correct number of words; TMT=trail making test.

### Reading span

ANCOVA tests showed significant relationships between SCC group and performance in the reading span task in accordance with the predictions. Individuals with a high level of SCC had poorer performance on both measures, i.e. span level, *F*(1, 211 )=12.85, *p*<0.001, and; total number of correctly freely recalled words across the test, *F*(1, 215 )=9.97 , *p*=0.002. 

The significant relationships between SCC group and the span level measure was reduced in effect size, but remained significant, when controlling for either, depressive symptoms (partial eta squared [η_p_^2^]= 0.022, *p*=0.030), or sleeping problems (η_p_^2^=0.026, *p*=0.019), while a trend remained when controlling for chronic stress symptoms. Controlling for these factors concomitantly reduced the effect size the most. For the free recall measure, controlling for either factor alone, or all factors concomitantly, decreased the relation between SCC group and free recall to non-significance, although a trend still remained after controlling for sleeping problems.

### 2-back

Results from the ANCOVA tests revealed that a high level of SCC was associated with significantly poorer performance on the 2-back target trials for all three types of measures: RT, *F*(1, 217 )=5.13 , *p*=0.025; accuracy, *F*(1, 217)= 4.37, *p*<0.038; ie, *F*(1, 217)=4.02 , *p*=0.046. No significant associations were seen between SCC group and the performance measures on the trials with new stimuli words, nor on trials with 3-back lure stimuli for which performance dropped to an equally low level in both SCC groups. 

Additionally controlling for either chronic stress, depressive symptoms or sleeping problems by adding these as covariates in the ANCOVA tests reduced the significant relationships between SCC group and performance on the 2-back target/hit trials on measures of accuracy, RT and IE to non-significance. However, when only controlling for sleeping problems, a trend remained for the relationship between SCC group and RT on the 2-back target trials. 

### TMT A and B

ANCOVA tests showed that the group with a high level of SCC had poorer (i.e. slower) performance on TMT part B, F(1, 119)=6.86, *p*<0.009; and also on the TMT B-A difference, *F*(1, 119)=7.67, *p*<0.006. No group differences were seen in performance on TMT part A.

Additionally, there were no group differences in response accuracy on either part A or B that needed to be adjusted for in the response time performance measures.

Additionally controlling for either chronic stress or depressive symptoms by adding these as covariates in the ANCOVA tests reduced the significant relationships between SCC group and response time on TMT B and on TMT B-A difference to non-significance. When controlling for sleeping problems the relationships between SCC and TMT B performance and the TMT B-A difference measure remained as a trend for the former and remained significant for the latter(η_p_^2^=0.020, *p*=0.041). 

None of the group differences on the measures of executive cognitive functioning remained significant after adjusting for chronic stress, depressive symptoms and sleeping problems simultaneously.

## Discussion

The present study tested the relation between SCC and executive functioning in a sample of the general working population, using three common tests tapping executive processes (i.e. two working memory tasks and an attentional shifting test). It was found that individuals with a high level of SCC performed significantly poorer on all three tests of executive cognitive ability, on those test measures that tap performance at a high executive load. Furthermore, on test measures of performance during low demands on executive processes (i.e. TMT part A, and “easy” trials with new stimuli words in the 2-back task), there were no differences in performance between individuals with high and low levels of SCC. This indicates that SCC was specifically associated with poorer ability to perform cognitive operations that place high demands on executive processes and may not be explained by generally poorer processing speed.

However, a high SCC level was not associated with poorer performance on the 3-back lure trials in the 2-back task, which also place high demands on executive processes. Instead, performance on 3-back lure trials deteriorated to an equally low level in both groups with high and low levels of SCC, possibly indicating that these trial types are at a level of difficulty where discriminability of any group differences in temporal updating processes decreases, as performance levels move toward floor effects.

The fact that the strongest relation was seen between SCC and WM span, may also suggest that SCC among employees may be more associated with specific executive deficits that are tapped by the WM span task used. WM span tasks as well as updating tasks require focused attention, and the maintenance of information in the face of distraction. Furthermore, both types of tasks seem to share important WM processes of building, maintaining, and updating arbitrary bindings [70,71].

However, the *extent* to which certain processes are tapped by the two types of tasks may differ. While n-back tasks tap updating processes, complex working memory span measures like the RS may tap WM *capacity* to a greater extent. This capacity has also been described as “the critical, executive-attention capability by which memory representations—for action plans, goal states, or environmental stimuli—are maintained in a highly active and easily accessible state” [72]. That is, the demands on temporary storage may be greater in the RS task than the 2-back task, as there are generally more items that need to be maintained simultaneously in each trial (2-5 items compared to only the last 2 items in the 2-back task). Furthermore, the RS task involves a second processing task that is performed in between presentations of the to-be-remembered items, such that additional attentional resources are needed to process this task and to resist interference of the second task with the to-be-remembered items. Thus, the RS task place greater demands on simultaneous short term storage as well as resistance to interference from the distracting task- in addition to demands on continuously updating (building and releasing) bindings, that is also required in the 2-back task.

Additionally, it has been argued that complex WM span task performance is determined by both the ability to maintain information accessible in primary memory and the ability to store and retrieve information from secondary memory [73]. With fewer items to be remembered simultaneously in the 2-back task, it may be argued that the RS task tap the ability to store and access information in secondary storage to a greater extent. 

The stronger relation between SCC and complex WM span thus suggest that SCC in the working population is most strongly associated with WM capacity, including the efficiency of related secondary memory processes.

The results are consistent with our own previous findings indicating that SCC among employees are related to poorer executive cognitive ability in a somewhat similar task with simultaneous demands on temporary storage and processing of a second task. That is, memory performance in verbal immediate free recall specifically during divided attention conditions (that place higher demands on executive cognitive attentional processes than do focused attention conditions) have been found to be poorer among individuals with a higher level of SCC, as compared to matched controls with little SCC (Stenfors et al. 2013, submitted manuscript/under review). 

Furthermore, the results are in line with some of the few studies by other research groups that have tested the relation between SCC and executive cognitive functioning among non-elderly adults. That is, Ruiz-Sanchez de Leon and colleagues tested and found the dysexecutive questionnaire to be related to performance on tests of memory, attention and executive function among persons with SCC that had contacted a memory clinic (n=50), compared to healthy controls (n=67) without SCC [20]. 

Reid and colleagues also found ratings on the memory complaint questionnaire among healthy aircraft maintenance personnel (n=866, ages 29-60 years) to be weakly associated to cognitive performance on the executive tests TMT B, digit symbol and the controlled oral word association test, but not on digit span forward and backward [19]. However, Scholtissen-In de Braek and colleagues reported no relation between SCC and measures of executive cognitive performance in a general population sample (n=499), using versions of the TMT A and B, colour-word stroop, and the letter digit substitution test in relation to ratings on the Maastricht Attention and Memory Checklist [2]. The fact that this sample was more heterogeneous regarding age and work status may partly explain the absence of relationships between subjective and objective measures of executive functioning. Our own sample on the other hand only included persons that were gainfully working and within the working age span (24-65 years) and thus were more homogenous regarding general living situation, which may affect the relationships between SCC and cognitive functioning . For example, it has been found that the relation between subjective and objective cognitive measures in elderly is moderated by differences in living situation in the sense that subjective self-ratings of cognitive functioning by persons that are more socially engaged and active are more strongly related to actual cognitive functioning than the self-ratings made by less socially active persons are [74]. This is likely due to the socially active people having both a relevant frame of reference for their own cognitive functioning level (i.e., it is possible to compare oneself in relation to others of similar age and socioeconomic status), as well as having their cognitive capacities challenged in daily life such that any cognitive difficulties can also be discovered. In a similar way, being active in modern working life should both provide a relevant frame of reference regarding ones cognitive capacities in relation to similar others, as well as provide continuous ecological “tests” of ones cognitive capacities when performing ones various work-tasks. Furthermore, modern work life increasingly requires the handling of multiple channels of information and interruptions, which clearly tap the executive cognitive resources. As such, any problems with executive functioning may be particularly noticeable, and problematic, among individuals in work life. This may partly explain why there seem to be a clearer relationship between SCC and executive functioning in the working part of the population.

Differences in findings between studies could also be attributable to other methodological differences, such as the efficiency of the subjective and/or objective cognitive measures used in capturing the intended construct and generating sufficient variability in order to detect individual differences present in levels of SCC and cognitive abilities.

For example, the measure of SCC used in the present study was based on global questions regarding cognitive function rather than questions about specific situations and cognitive errors that are commonly used in other self-rating instruments for subjective cognitive functioning. For example, questions regarding specific situations concerning memory were used in Reid et al.’s study [19], and a mix of more specific and more global questions concerning attention and memory were used in the study by Scholtissen-In de Braek et al. [2], which may play a role when making comparisons between study findings.

While SCC may relate more to how a person experience that he or she is functioning cognitively in real life, objective cognitive function on the other hand is assessed in controlled and structured settings. Although the differences in WM and shifting performance that were found between individuals with high and low levels of SCC in the present study are small in terms of absolute scores and effect size, they may represent a cognitive difference of significance in every-day life. That is, even small differences in executive performance during controlled conditions could in real life settings make a significant difference to how well a person can handle demanding situations and tasks. For example, having less executive resources (such as lower WM capacity to maintain information active in awareness) will also likely involve a higher vulnerability to fatigue/resource depletion of these limited cognitive resources. This type of resource depletion can occur for example from extra cognitive demands that commonly arise in ecological settings, such as the need to resist interference from external and internal distractors that are threatening the focus of attention on those cognitive operations that are relevant for the present task an individual is trying to perform. This means that handling a working environment or work demands that place particularly high demands on executive functioning may become more troublesome for individuals with poorer executive cognitive function and more SCC. As such, the experience of SCC could potentially be exacerbated by contexts that are particularly demanding on the limited executive resources, and individuals with poorer actual executive ability are then likely to be more vulnerable to negative effects on subjective and objective cognitive performance. This is also indicated by the previous finding, mentioned above, that employees with more SCC are more vulnerable to declines in memory performance when faced with distraction from a secondary task, than are those employees that experience little SCC (Stenfors et al., 2013, submitted manuscript/under review). Furthermore, SCC in the work force have been found to be associated with having a high work load and having high demands related to information and communication technology (e.g. being interrupted by and having to respond to too many phone calls and e-mails) suggesting that certain work characteristics may increase the experience of SCC [3].

The present findings suggest that SCC among employees may be handled via interventions both at the individual level that target executive functioning, as well as at the level of the work organisation, work environment design and work etiquette, such that unnecessary strain on employees’ executive cognitive resources are minimized. 

Symptoms of depression, chronic stress and sleeping problems overlap with SCC and the groups with high and low SCC differ significantly in the level of those symptoms. Particularly symptoms of chronic stress and depression were found to play a role in the relationships between SCC and executive functioning also in the present study, in line with similar findings by others’ [19]. However, an independent relationship remained between SCC and working memory capacity for one of the WM measures (i.e. span level on the reading span test) even after adjustment for symptoms of depression or chronic stress, suggesting that either of these symptoms or conditions may not fully explain the relationship between SCC and WM capacity. When adjusting for both types of symptoms simultaneously, as well as sleeping problems, the relation between SCC and WM span was no longer significant. Thus, the lower WM span, as well as poorer WM performance on the 2-back task and shifting on the TMT, that was found to be related to higher SCC may be explained by these other psychological health problems or by a third factor related to all these measures, among individuals in the working population. However, applying these extensive adjustments to the analyses also introduces a risk of over adjustment. Due to this, and the cross sectional design, it cannot be concluded that these other psychological symptoms are the causes of the SCC and related executive functioning deficits seen in this study. Rather, there are several possibilities as to how these symptoms are inter-related in the working population. SCC and executive cognitive problems may be part of, and secondary to, depressive and stress-related conditions among employees, as executive cognitive functioning is also known to be implicated in these conditions. In this case, SCC may be prevented or treated via prevention and treatment of these conditions. However, reduced executive functioning can also reduce the capacity to cope with stressors and regulate emotions and thoughts, and may make individuals more vulnerable to depressive and stress-related symptoms [24,75,76]. As such, there may be multiple and bi-directional causal relationships between all these problems and thus multiple strategies of preventing and treating SCC may be most effective, at the individual level as well as the organisational and work place level. 

Although other psychological problems are present where SCC is related to poorer executive functioning, asking individuals about SCC can provide additional information regarding their cognitive status. 

### Strengths and limitations

The sample of participants was drawn from a larger cohort study that is representative of the general Swedish working population, making the results generalizable among employees and not being limited to specific clinical groups or work places. The control participants were also matched to the cases, who reported a high level of SCC, on important variables affecting SCC and cognitive functioning level. However, since study cases were invited to participate based on their reported levels of SCC, and as high levels of SCC are approximately twice as common among women compared to men in the work force, this is also reflected in the gender distribution among the study participants. The results may thus be more representative for employed women than men. 

The cross sectional design does not allow inferences to be made regarding causal relationships between other psychological symptoms- i.e. symptoms of depression and chronic stress- and SCC and executive functioning.

## Conclusions

The present study suggests that higher levels of SCC are related to poorer executive cognitive functioning among working non-elderly adults. Thus, policies and interventions at the level of health care, management, the work place, and individual strategies that strengthen and protect the individual’s executive cognitive resources may be particularly relevant and helpful to employees presenting with SCC. 

Symptoms of stress, depression and sleeping problems should be attended to in interventions for SCC as these co-occur with- and may play a role in SCC and associated executive cognitive problems.

The present study contributes to increased understanding of SCC in the work force. The findings may be particularly relevant as many modern jobs and work environments place high demands on the executive cognitive capacities of the employed, and since psychological problems that often involve cognitive complaints currently constitute the greatest cause of sick-leave in Sweden. At the same time, the work force is becoming older and need to stay working for longer. 
